# Hemorrhagic Fever Viruses: Pathogenesis and Countermeasures

**DOI:** 10.3390/microorganisms10030591

**Published:** 2022-03-09

**Authors:** Chad E. Mire, Andrea Marzi

**Affiliations:** 1 Galveston National Laboratory, Department of Microbiology and Immunology, University of Texas Medical Branch, Galveston, TX 77555, USA; chmire@utmb.edu; 2 Laboratory of Virology, Division of Intramural Research, National Institute of Allergy and Infectious Diseases, National Institutes of Health, Hamilton, MT 59840, USA

Before December 2019 and the COVID-19 pandemic, the general public was to some extent aware that zoonotic viruses can spill over into the human population and cause a disease outbreak. This is due, in part, to the unprecedented Ebola virus (EBOV) epidemic from 2013–2016 in West Africa receiving heavy global media attention. Hemorrhagic fever viruses (HFVs) have had sporadic documented outbreaks over the past five decades with an increase in these events over the last twenty years. Perhaps the increase in documented HFV outbreaks is due to lessons learned over the past five decades along with advanced diagnostic tools and coordinated international responses that helped build “in-country” capacity to increase surveillance for the known hemorrhagic fevers where these viruses are endemic. This leads to the question as to whether we are experiencing more viral hemorrhagic fever outbreaks or if we have improved at identifying them. Regardless of the answer(s) to this question, research on HFVs is an important endeavor to understand their impact on humans in regard to disease, transmission, epidemiology, ecology, diagnostics, therapeutics, and vaccines. This Special Issue “Hemorrhagic Fever Viruses: Pathogenesis and Countermeasures” includes 19 manuscripts on tick-borne viruses, arenaviruses, and filoviruses covering the following topics: epidemiology, assay development, molecular virology, animal models, vaccines, and treatment strategies.

The range and reach of viruses will most certainly shift as climate and land use change with a potential large impact on the range of tick-borne hemorrhagic fever viruses. In this special issue, novel insights into tick-borne hemorrhagic fever viruses are discussed in order to understand what is currently occurring in endemic areas for these viruses.

Crimean-Congo Hemorrhagic Fever virus (CCHFV) already has a large distribution for a tick-borne virus which spans across Africa, Europe, and Asia. Wampande et al. present data on the CCHFV phylogenetics of isolates from African blue ticks found on cattle in Uganda [1]. These data led to the discovery of a new CCHFV strain within the African genotype II clade which will aid in understanding circulation of the virus in ticks and within Africa to inform medical counter measures (MCMs) and diagnostics. Beyond CCHFV in Africa, Portillo et al. present insights into the epidemiology of a relatively new area for CCHFV in Western Europe in particular the ticks and animals where the virus, through different means, has been detected in Spain [2]. To further understand the important genes for CCHFV to replicate in mammals, Welch et al. provide data for CCHFV infection of mice with a recombinant virus lacking the complete NSm gene demonstrating that this protein is dispensable for growth in mice [3]. Working with CCHFV requires biosafety level 4 (BSL4) laboratories whereas closely related BSL2 viruses such as Hazara orthonairovirus (HAZV) offer an alternative model to study CCHFV. Hartlaub et al. attempted to establish infections in sheep and cattle to compare HAZV to CCHFV infection in these animals and found that infection and serology were distinct from CCHFV [4]. The final manuscript covering tick-borne viruses in this special issue is a comprehensive review of two flaviviruses found in India (Kyanasur Forest disease virus (KFDV)) and Saudi Arabia along with Egypt (Alkurma hemorrhagic fever virus (AHFV)) highlighting the expansion of the endemic areas of these viruses [5]. These flaviviruses are BSL4 pathogens with case fatality rates (CFRs), warranting attention as public health concerns.

Covered in this special issue is Lassa fever virus (LASV), an arenavirus with CFRs of public health concern that is associated with hemorrhagic fever outbreaks in Africa such as the one currently ongoing in Nigeria [6]. Shaffer et al. present data assessing the serology of patients presenting with hemorrhagic fever symptoms in Sierra Leone from 2012–2019 to understand the incidences and locations of Lassa fever (LF) during that seven-year span [7]. Treatment of LF is an important way to manage outbreaks of LASV and Hansen et al. provide a comprehensive review of the current treatment strategies and therapeutics that have shown promise in preclinical animal models and discuss their potential to move into clinical trials with the possibility of approval for human use [8]. Beyond what is known for the LF treatment pipeline, Sahin et al. present novel data on the use of the Janus kinase inhibitor Ruxolitinib in an arenavirus mouse model using lymphocytic choriomeningitis virus (LCMV) as a surrogate to understand the treatment benefit of IFNγ host-directed treatment which showed promise as an Arenavirus disease treatment [9]. As highlighted here and in several contributions to this special issue, animal models are key to our initial evaluation of antivirals and Condrey et al. report on key validations of clotting factors in LASV infection in Strain 13/N guinea pigs [10]. These data are not only important for LASV disease models but can also inform preclinical models of other HFVs such as filoviruses, which are also covered in this special issue.

Filoviruses are HFVs of public health concern associated with deadly outbreaks originating in Africa, such as EBOV, with the exception of Lloviu virus (LLOV), Reston virus (RESTV), and Taï Forest virus (TAFV) which have not been associated with high CFRs (TAFV) or have so for not been causing hemorrhagic disease in humans (LLOV, RESTV). While not associated with deadly outbreaks, these three filoviruses are still important to study and expand upon our knowledge why they are less pathogenic and/or their potential to cause a deadly outbreak. This special issue features manuscripts describing the entry receptor usage of LLOV [11], a manuscript investigating the potential reasons for RESTV attenuation [12], and a manuscript reporting on the lack of lethality of TAFV infection in ferrets [13] which differs from the lethal EBOV disease ferret model [14]. The high CFRs documented with EBOV infection attracts the attention of investigators; this virus is therefore heavily studied, a fact reflected in the number of EBOV manuscripts in this special issue. Detection of EBOV during outbreaks is an important part of outbreak response and Furuyama and Marzi describe an ELISA to detect EBOV soluble glycoprotein (sGP) as a diagnostic tool for early detection of EBOV infection [15]. Understanding virus load is an important tool used to assess vaccines and treatments though laboratories from different institutions use either focus forming unit assay, plaque assay, or 50% tissue culture infectious dose (TCID_50_) assays and Keiser et al. compare and contrast these different assays [16]. These assays can be useful when comparing treatments in animal models of EBOV disease such as the “gold standard” nonhuman primate (NHP) models. Alfson et al. discuss data to characterize the rhesus macaque NHP model to support MCMs for EBOV disease [17]. The EBOV NHP model is further highlighted in this special issue through cynomolgus macaque transcriptomics and host miRNAs data that are associated with fatal disease outcome [18]. In depth understanding of pathogenesis through these models can often lead to information that can be used to approach how to treat these diseases using previously approved drugs [19], how to deploy vaccines that are efficacious [20], and how routine care in EBOV intensive care units can present risks to health care workers from patients [21].

In summary, this special issue “Hemorrhagic Fever Viruses: Pathogenesis and Countermeasures” covers epidemiology, assay development, molecular virology, animal models, vaccines, and treatments for HFV including tick-borne viruses, arenaviruses, and filoviruses. Each manuscript in the issue adds an important aspect to the HFV field which will aid future research and outbreak response.
